# Delta radiomics analysis for prediction of intermediary- and high-risk factors for patients with locally advanced cervical cancer receiving neoadjuvant therapy

**DOI:** 10.1038/s41598-023-46621-y

**Published:** 2023-11-08

**Authors:** Rong-Rong Wu, Yi-Min Zhou, Xing-Yun Xie, Jin-Yang Chen, Ke-Run Quan, Yu-Ting Wei, Xiao-Yi Xia, Wen-Juan Chen

**Affiliations:** 1 Department of Radiation Oncology, Department of Gynecology, Clinical Oncology School of Fujian Medical University, Fujian Cancer Hospital, Fuzhou, China; 2 School of Nuclear Science and Technology, University of South China, Hengyang, China; 3 College of Computer and Cyber Security, Fujian Normal University, Fuzhou, China; 4 Department of Radiation Oncology, Xiangtan City Central Hospital Xiangtan, Hengyang, China

**Keywords:** Cancer, Cancer genomics, Gynaecological cancer

## Abstract

This study aimed to assess the feasibility of using magnetic resonance imaging (MRI)-based Delta radiomics characteristics extrapolated from the Ax LAVA + C series to identify intermediary- and high-risk factors in patients with cervical cancer undergoing surgery following neoadjuvant chemoradiotherapy. A total of 157 patients were divided into two groups: those without any intermediary- or high-risk factors and those with one intermediary-risk factor (negative group; n = 75). Those with any high-risk factor or more than one intermediary-risk factor (positive group; n = 82). Radiomics characteristics were extracted using Ax-LAVA + C MRI sequences. The data was divided into training (n = 126) and test (n = 31) sets in an 8:2 ratio. The training set data features were selected using the Mann–Whitney *U* test and the Least Absolute Shrinkage and Selection Operator (LASSO) test. The best radiomics features were then analyzed to build a preoperative predictive radiomics model for predicting intermediary- and high-risk factors in cervical cancer. Three models—the clinical model, the radiomics model, and the combined clinic and radiomics model—were developed in this study utilizing the random forest Algorithm. The receiver operating characteristic (ROC) curve, decision curve analysis (DCA), accuracy, sensitivity, and specificity were used to assess the predictive efficacy and clinical benefits of each model. Three models were developed in this study to predict intermediary- and high-risk variables associated with postoperative pathology for patients who underwent surgery after receiving neoadjuvant radiation. In the training and test sets, the AUC values assessed using the clinical model, radiomics model, and combined clinical and radiomics models were 0.76 and 0.70, 0.88 and 0.86, and 0.91 and 0.89, respectively. The use of machine learning algorithms to analyze Delta Ax LAVA + C MRI radiomics features can aid in the prediction of intermediary- and high-risk factors in patients with cervical cancer receiving neoadjuvant therapy.

## Introduction

Cervical cancer is the fourth most commonly diagnosed cancer in women globally. Approximately 84% of cases are detected in underdeveloped countries, where cervical cancer is the leading cause of cancer-related mortality in women. Cervical cancer is treated with surgery, radiation, and chemotherapy^1^. Friedlander^2^ defined a "neoadjuvant chemotherapy" (NACT) in 1983 to describe two to three cycles of systemic chemotherapy administered to patients with malignant tumors before surgery or radiation therapy to reduce the tumor. In stage Ib2-IIb cervical cancer (FIGO 2009 version), direct surgical resection is difficult due to the large tumor size. NACT is typically employed to shrink the tumor and improve the surgical resection rate^3^. This strategy is not only beneficial for the surgical treatment of patients with inoperable cancer but also provides the opportunity to preserve the ovaries of young patients. NACT can reduce tumor cell viability and eliminate micro-metastases, which can help mitigate postoperative high-risk pathological factors, reduce postoperative radiotherapy rates, and improve the postoperative quality of life of patients^4, 5^. Hence, NACT followed by surgery has been rendered an alternative radical treatment modality for patients with locally advanced cervical cancer (LACC), in addition to concurrent chemoradiotherapy^6^. While the NCCN guidelines do not endorse neoadjuvant treatment options, the 2016 American Society of Clinical Oncology Resource Stratification Clinical Practice Guideline^7^ emphasizes the reliance on alternative resources for treatment when radiation therapy is unavailable in certain situations. Instead of surgery followed by chemotherapy, this panel favors neoadjuvant chemotherapy. Neoadjuvant chemotherapy followed by surgery is a successful treatment option for patients with locally advanced cervical cancer in resource-limited settings.

In LACC, cisplatin-based concurrent radiation has a better the disease-free survival (DFS) rate than NACT followed by radical surgery; however, no statistically significant differences are observed in the overall survival (OS) rates^8^. NACT followed by radical surgery has demonstrated an OS rate similar to that observed with concurrent radiation in patients with stage IB3 and IIA2 cervical cancer, with no increase in side effects^9^. In patients with comparable survival, approximately 25–30% of patients who undergo surgery after NACT still require adjuvant radiotherapy or chemoradiotherapy. This treatment model has potential issues such as increased medical costs, treatment-related diseases, myelosuppression, lymphedema, urinary and rectal fistulas, rectal strictures, bladder dysfunction, and rectal stenosis^10^.

Through non-invasive manipulation, radiomics may aid in extracting many radiomics features and obtaining tumor heterogeneity information. Researchers can mine high-dimensional data that can aid in therapeutic decision-making, forecast tumor survival, and, ultimately, help in personalized and precise treatment^11^. Radiomics has been applied to lung cancer, breast cancer, cervical cancer, and so on^12–14^.

Tian et al.^4^ used computed tomography (CT)-based radiomics to predict response to NACT in patients with LACC in a multicenter clinical investigation. Surgery after NACT was conducted for patients in the investigation cohort; individuals with poor responses were advised to receive direct concurrent chemoradiotherapy. Many scholars have used radiomics to predict risk factors marking the postoperative period in patients with cervical cancer^15–18^. However, intermediary- and high-risk factors have not been predicted in patients who undergo surgery after NACT. Giannini V, Jeon SH, and van Dijk LV et al. predicted response to tumor therapy using Delta radiomics features before and after treatment^19–21^. Therefore, in this study, we aimed to use Delta radiomics features extracted before and after neoadjuvant therapy to predict intermediary- and high-risk postoperative parameters in this patient cohort.

## Materials and methods

### General materials

This study retrospectively collected data corresponding to 157 patients (cervical cancer: stage IIA2 ~ IIB) who underwent radical cervical cancer surgery after completion of neoadjuvant therapy at Fujian Cancer Hospital from January 2013 to July 2018. The 2009 FIGO staging system was used. Age, squamous cell carcinoma antigen level pre-treatment (pre-treatment SCC), after-treatment squamous cell carcinoma antigen level (after-treatment SCC), pre-treatment Hemoglobin (pre-treatment HB), maximum tumor diameter, Tumor volume before treatment, Tumor volume after treatment, Tumor volume regression rate, Recist v. 1.1 tumor regression rate and WHO tumor regression rate were collected to the clinical data.

Inclusion criteria: (1) Patients who had not been subjected to any treatment outside our hospital premises during the first visit; (2) pelvic magnetic resonance imaging (MRI) plain scan + enhancement examination was performed before and after neoadjuvant treatment; (3) radical hysterectomy and pelvic ± paraaortic lymphadenectomy; (4) preoperative scan sequences of the patients included lower abdominal MRI Ax_LAVA + C sequences; (5) pathological results were clear after surgery.

Exclusion criteria: (1) artifacts in the imaging area of interest; (2) Ax_LAVA + C sequence images of MRI did not meet the requirements of tumor image segmentation; (3) incomplete clinical data; (4) patients who had not completed neoadjuvant treatment and surgery at our institution.

After screening based on inclusion and exclusion criteria, according to Sedlis Standard, a total of 157 patients were divided into two groups: those without any intermediary- or high-risk factors and those with one intermediary-risk factor (negative group; n = 75). Those with any high-risk factor or more than one intermediary-risk factor (positive group; n = 82). High-risk and intermediate-risk postoperative risk factors for cervical cancer are separated. Positive lymph nodes, paracervical infiltration, and positive margins are examples of high-risk factors. Vascular embolism, nerve invasion, tumor size, interstitial infiltration, and lymphovascular interstitial infiltration are intermediate risk factors. The patients were randomly assigned to the training and test sets in a ratio of 8:2, with 126 patients in the training set and 31 patients in the test set.

### Examination methods

All MRI examinations were performed using 1.5 T scanners (Signa 1. 5 T EXCITE III HD) with eight-channel phased-array abdominal coils. All patients were injected with the contrast agent gadopentetate glucosamine injection (Gd-DTPA) at a dose of 0.1 ~ 0.2 mmol/kg via elbow vein at a rate of 1.5 ml/s. The following criteria were used: TR was 4 ms, TE was 2 ms, the flip angle was 15°, FOV was 400 mm × 400 mm, layer thickness/interlayer distance = 7 mm/3.5 mm, number of layers was 88–92, and scanning time was about 15 s.

### Image segmentation and feature extraction

**Figure 1:**
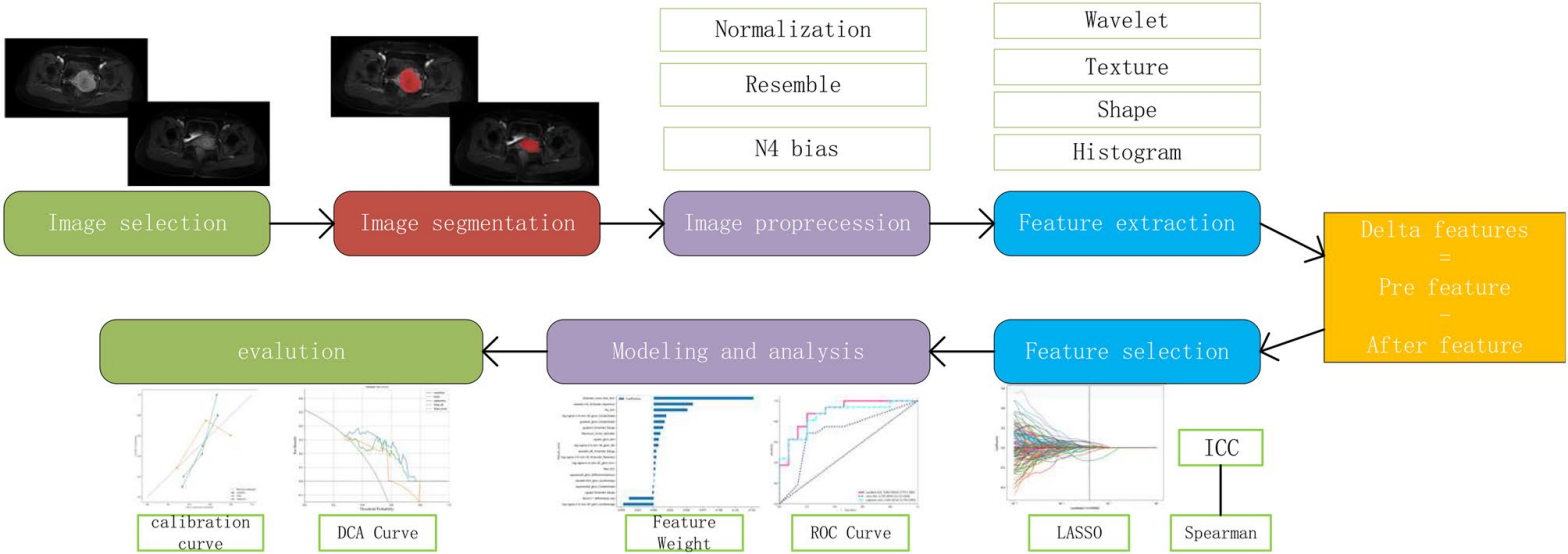
The workflow of radiomics analysis in this study.

To achieve grayscale normalization, Ax-LAVA + C images of each patient before and after neoadjuvant treatment were first subjected to N4-bias field correction. The corrected images were imported into the ITK-SNAP 3.8 software (www.itksnap.org). A gynecologic oncologist with five years of experience (physician 1) outlined all images layer-by-layer to fuse the region of interest into volume of interest (VOI). These images were then reviewed by a gynecologic oncologist with more than ten years of experience (physician 2). Image preprocessing was performed to enhance radiomics feature differentiation, including resampling to 1 × 1 × 1 mm isotropic voxels, and interpolation was performed using the SitkBSpline algorithm. Radiomics features were extracted by the Pyradiomics package. A total of 1562 radiomics features were extracted, including first-order histogram features, morphological features, texture features, and Gaussian wavelet transform filter features. The delta radiomics features were calculated based on the following formula: post-treatment radiomics features—pre-treatment radiomics features^22^. A total of 30 patients were selected using a completely randomized method, and the VOI was outlined again by physician 1 at one-month intervals. The intraclass correlation coefficient (ICC) consistency was evaluated by extracting the radiomics features^23^. The workflow of radiomics analysis was shown in Fig. 1.

### Feature selection and development of radiomics labels

First, the data were regularized, and the radiomics features in the training set were initially screened based on ICC (ICC > 0.75) to retain the features with good reproducibility. Second, after excluding highly redundant radiomics features with Spearman correlation coefficients > 0.9, radiomics features were assessed using the Mann–Whitney *U* test to distinguish between positive and negative groups while keeping statistically significant features. Third, the LASSO was used to perform feature dimensionality reduction. Finally, we used the random forest machine learning method to distinguish between positive and negative sets. All feature screening and classification operation procedures were performed with Python 3.8.5. A pre-and post-neoadjuvant delta radiomics model, a clinical model, and a pre-and post-neoadjuvant delta clinical radiomics model were constructed. All models were quantified using the ROC curve^24^, and the AUC values were used to quantify the predictive value of models. Accuracy, sensitivity, and specificity were also used to assess the diagnostic performance of models.

### Clinical benefits

The decision curve analysis (DCA) was used to determine the net benefit of each model for the evaluation of intermediary- and high-risk factors at various threshold probabilities and to rate the clinical usefulness of the predictive model^25^ .

### Statistical methods

All aforementioned statistical methods were performed via Python 3.8.5 and SPSS 26.0 (IBM, New York, USA).

### Ethics statement

This study was approved by the medical ethical committee review board of the Fujian Cancer Hospital (No. K2022-135-01).

## Results

### Comparison of baseline characteristics of patients in the two groups

Table 1 displays the clinical characteristics of the patients. The average age of the patients in the training set was 51.86 ± 7.41 years. The average age of the patients in the test group was 53.68 ± 7.56 years. Only the tumor regression rate determined via Recist v1.1 criteria^26^ and pre-treatment HB levels (p > 0.05) were significantly different between the two cohorts.

**Table 1:** Comparison of clinical features in the training and validation sets.

Clinical features	Training set	Test set	P
Tumor volume before treatment	31.20 ± 22.04	32.35 ± 29.74	0.809
Tumor volume after treatment	8.53 ± 8.59	8.26 ± 6.99	0.872
Tumor volume regression rate	0.70 ± 0.23	0.65 ± 0.25	0.334
Maximum tumor diameter before treatment	4.28 ± 1.19	3.99 ± 1.59	0.242
Maximum tumor diameter after treatment	1.80 ± 1.43	1.39 ± 1.21	0.145
WHO tumor regression rate	0.59 ± 0.31	0.63 ± 0.35	0.583
Recist v1.1 tumor regression rate	0.31 ± 0.35	0.47 ± 0.39	0.025
Pre-treatment HB	110.30 ± 18.14	118.48 ± 18.90	0.021
Pre-treatment SCC	8.66 ± 11.37	8.86 ± 12.71	0.443
after-treatment SCC	2.75 ± 4.23	2.73 ± 4.92	0.985
Age	51.86 ± 7.41	53.68 ± 7.56	0.224
Diameter			0.091
< 4 cm	53(42.06%)	12(38.71%)	
≥ 4 cm	73(57.94%)	19(61.29%)	

### Feature extraction and feature dimensionality reduction

**Figure 2:**
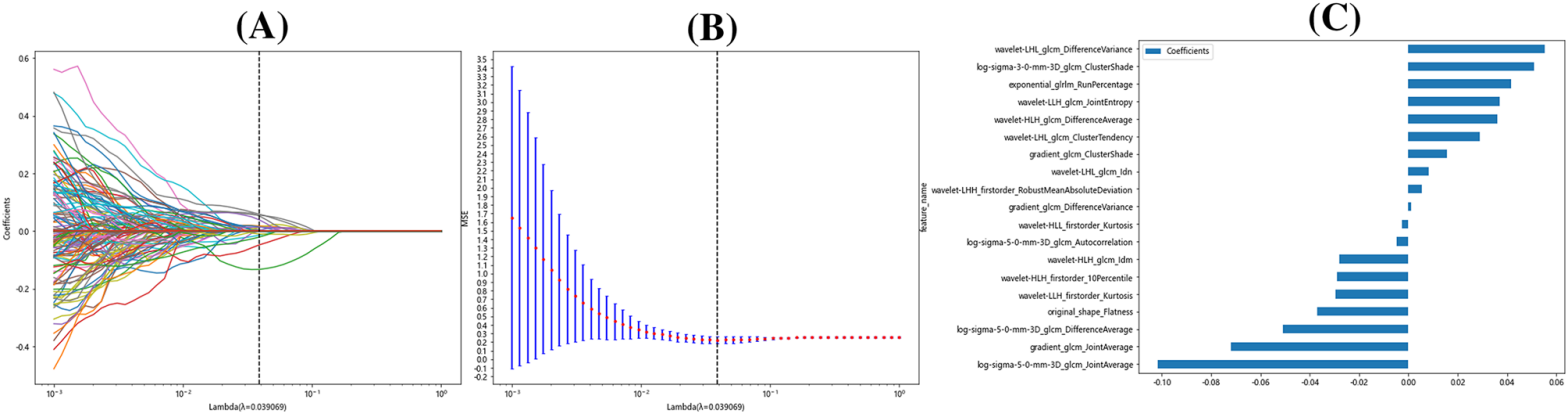
(**A**) Coefficient profiles. (**B**) cross-validation plot. (**C**) feature weight plot.

We extracted 1562 radiomics features from Ax LAVA + C MRI sequence images captured before and after neoadjuvant therapy. The delta radiomics features were calculated. A completely random process was used to select 30 patients, and physician 1 again marked the VOI after an interval of one month. Following the extraction of radiomics features, consistency in the ICC was assessed. Among all extracted radiomics features, features with ICC > 0.75 were selected. For identifying redundant features, Spearman correlation coefficients were calculated. One feature was arbitrarily excluded if the correlation coefficient between two features was greater than 0.9. Then, the best subset of radiomics features was selected using the LASSO test based on tenfold cross-validation for penalty adjustment. The features retained in the model are shown in Fig. 2.

### Construction of clinical model

**Figure 3:**
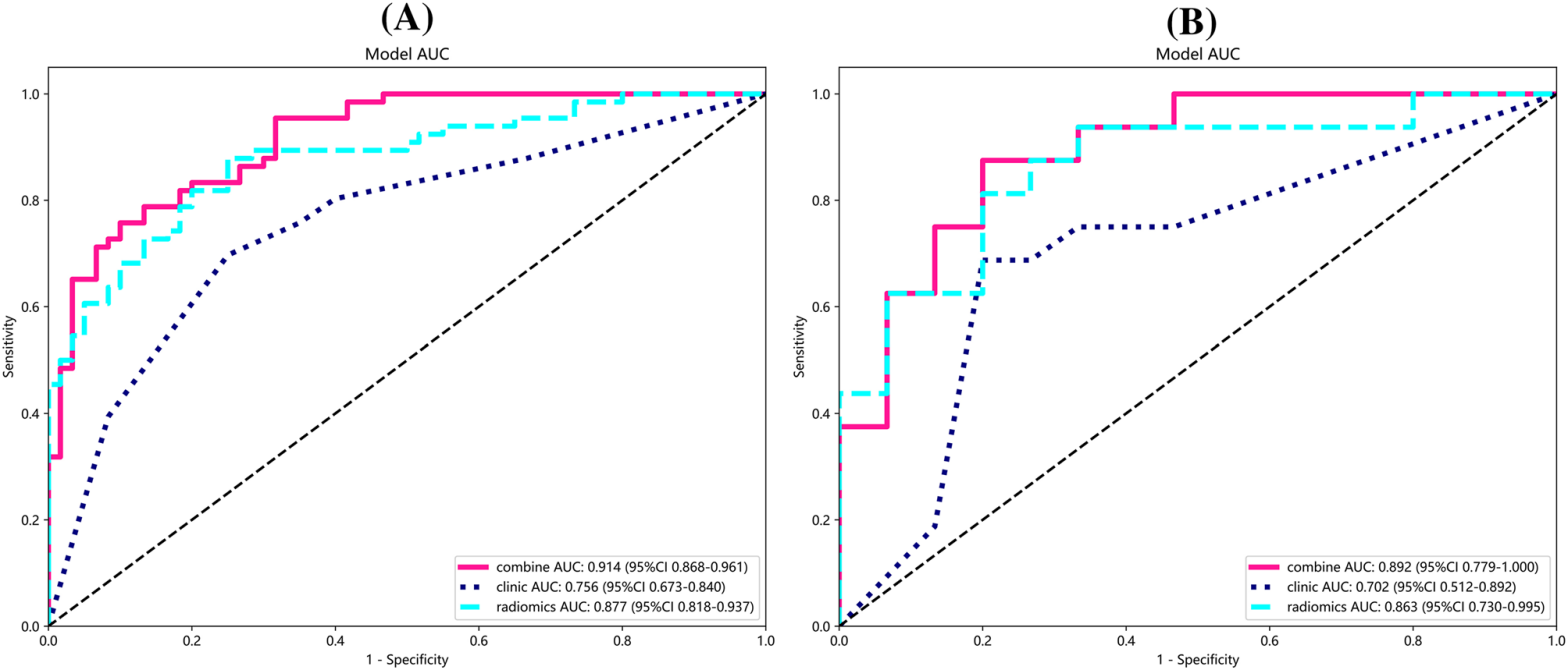
(**A**) The ROC curves of the clinical model, radiomics model, and combined model in the training set. (**B**) The ROC curves of the clinical model, radiomics model, and combined model in the test set.

We retained the following 7 clinical features through feature selection to construct a clinical model: Age, Diameter, Pre-treatment SCC, Tumor volume after treatment, after-treatment SCC, WHO_withdrawal_rate and Recist1.1_withdrawal_rate. The clinical model was constructed by applying the random forest algorithm based on clinical features with an AUC of 0.76 (95% CI 0.673–0.840) in the training set with precision, sensitivity, and specificity of 0.72, 0.70, and 0.82, respectively, and an AUC of 0.74 (95% CI 0.512–0.892) in the test set with precision, sensitivity, and specificity of 0.74, 0.69, and 0.92, respectively (Fig. 3).

### Construction of radiomics model

The model was developed with delta radiomics features calculated based on Ax_LAVA + C MRI sequences before and after neoadjuvant treatment. A total of 18 radiomics features were retained after feature extraction and feature dimensionality reduction (Additional file 1). The random forest algorithm was applied to construct a radiomics model based on the above features. The AUC in the training set was 0.88 (95% CI 0.818–0.937), and the precision, sensitivity, and specificity were 0.82, 0.88, and 0.75, respectively. The AUC in the test set was 0.86 (95% CI 0.730–0.995), and the precision, sensitivity, and specificity were 0.81, 0.81, and 0.80, respectively (Fig. 3).

### Development of combined clinical radiomics model

Based on Ax_LAVA + C MRI sequences collected before and after neoadjuvant therapy, delta radiomics features were calculated. From clinical features and delta radiomics features combined, 4 clinical features and 15 radiomics features were retained after feature extraction and feature dimensionality reduction (Additional File 1). The random forest algorithm was applied to construct a model based on the aforementioned features, with an AUC of 0.91 (95% CI 0.868–0.961) in the training set and accuracy, sensitivity, and specificity of 0.83, 0.76, and 0.90, respectively. The AUC in the test set was 0.89 (95% CI 0.779–1.000), and the precision, sensitivity, and specificity were 0.84, 0.88, and 0.80, respectively (Table 2).

**Table 2:** Performance of each model.

Model name	Data set	AUC	95% CI	Accuracy	Sensitivity	Specificity	Threshold
Radiomics model	Training set	0.88	0.818–0.937	0.82	0.88	0.75	0.516
	Test set	0.86	0.730–0.995	0.81	0.81	0.80	0.533
Clinic model	Training set	0.76	0.673–0.840	0.72	0.70	0.82	0.578
	Test set	0.70	0.512–0.892	0.74	0.69	0.92	0.578
Combine model	Training set	0.91	0.868–0.961	0.83	0.76	0.90	0.590
	Test set	0.89	0.779–1.000	0.84	0.88	0.80	0.557

### Clinical benefits

**Figure 4:**
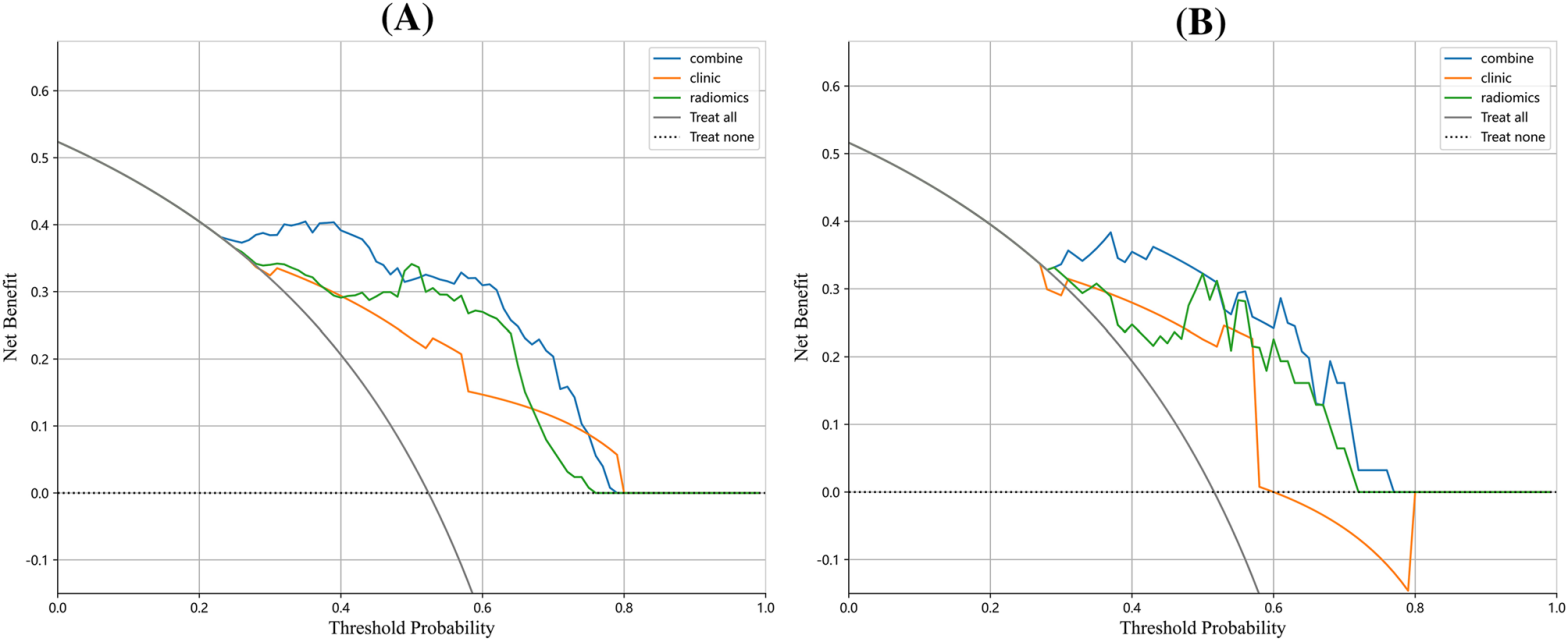
(**A**) The DCA curves in the training set. (**B**) The DCA curves in the test set.

The findings of decision curve analysis (DCA) for the clinical, radiomics, and combined models are shown in Fig. 4. The decision curves showed that the combined model performed better in terms of benefits than the clinic model and radiomics model.

## Discussion

Cervical cancer is one of the most common malignant tumors in gynecology, and its incidence rate is second only to breast cancer, with the third highest mortality rate in developing countries^27^. According to the World Health Organization, over 80% of cervical cancer cases occur in developing nations. Additionally, more than half of cervical cancer patients are diagnosed at an advanced stage. Cervical cancer exhibits a subtle onset with inconspicuous early symptoms, and screening and treatment techniques for the disease vary across countries and regions. In cases where lesions exceed 4 cm, the likelihood of recurrence and metastasis after treatment increases for stage IB2 and IIA2 cervical cancer. Despite the NCCN guidelines recommending concurrent chemoradiotherapy as the preferred treatment approach, the 5-year survival rate for patients undergoing this treatment ranges from approximately 30% to 80%^28^. In certain regions, there is a low number of patients with stage IB2 and IIA2 cervical cancer and large lesions receiving CCRT due to limitations in radiotherapy equipment and techniques. In some areas, stage IIB patients also undergo surgery as a treatment option. Apart from the constraints related to radiotherapy equipment and technology in certain developing countries, such as China, the decision-making process of patients and their families also plays a role in the selection of treatment approaches. The optimal treatment modality for this specific group of patients is still under exploration. Neoadjuvant chemotherapy can effectively decrease the requirement for postoperative adjuvant radiotherapy and enhance the postoperative quality of life for young women and patients with locally advanced cervical cancer and large lesions. Its primary objective is to preserve the ovarian and sexual functions of these young patients without compromising their prognosis. Therefore, accurately predicting the effectiveness of neoadjuvant therapy before treatment and identifying intermediary- and high-risk factors after surgery are essential factors for this specific group of patients.

In this study, a clinical and radiomics model and a combined clinical radiomics model were constructed to predict intermediary- and high-risk factors associated with postoperative pathology after neoadjuvant therapy. The models were based on delta radiomics features and clinical features of Ax_LAVA + C MRI sequence images collected before and after neoadjuvant therapy. Delta radiomics includes a temporal component, including the extraction of quantitative features from picture sets recorded during therapy, which reveals the progression of feature values. The combined clinical radiomics model showed the best predictive ability with respect to both the training set (AUC: 0.91) and the test set (AUC: 0.89). Nonetheless, the radiomics model outperformed the clinical model in this study (radiomics model AUC of 0.86 vs. clinical model AUC of 0.70 in the test set). The combined clinical radiomics model showed the best predictive performance with AUC, accuracy, and sensitivity of 0.89, 0.84, and 0.88, respectively, in the test set (Fig. 3).

Yang-yang Kan et al.^16^ previously used T1CE and T2 MRI radiomics to identify positive lymph nodes in patients with early-stage cervical cancer, with AUC values of 0.753 (95% CI 0.656–0.850) in the training set and 0.754 (95% CI 0584–0.924) in the test set. MRI radiomics were used to predict lymphovascular interstitial infiltration in patients with early-stage cervical cancer, as well as risk stratification for patients who required adjuvant radiotherapy after surgery^29^; Sun et al.^5^ believed that before treatment, MRI-based radiomics predicts LACC NACT responses. With an AUC of 0.999, the combined model outperformed other radiological models in the test set. Fang et al.^26^ showed that a radiomics model was developed to predict the response to simultaneous radiotherapy using three sequences of sagittal T2 images, ADC map images, and axial T1-enhanced images obtained during pre-treatment multiparametric MRI. The AUC values were 0.820 (95% confidence interval 0.713–0.927) and 0.798 (95% confidence interval 0.678–0.917) in the training and the test sets, respectively. Furthermore, the combined model outperformed individual radiomics or clinical features, consistent with the findings of the current study. Our study is novel in that it proposes predicting intermediary-and high-risk factors for patients after neoadjuvant therapy in the postoperative period to assist clinicians in determining whether adjuvant therapy is needed or whether concurrent radiotherapy should be administered directly.

There are certain limitations to this study that need to be addressed. Firstly, there might be a selection bias since all the data used in this study were obtained from a single study center and a specific type of MRI machine. Future research should include validation from multiple centers to ensure generalizability. Secondly, the study participants were limited to cervical squamous carcinoma, and it would be beneficial to conduct studies involving other pathological subtypes of cervical cancer.

In summary, a combined clinical radiomics model was developed using Delta LAVA + C MRI radiomics features and clinical features. Machine learning algorithms are expected to be used for non-invasive preoperative prediction of intermediary- and high-risk parameters in patients with cervical cancer receiving neoadjuvant therapy.

## Conclusion

In conclusion, we established an MRI-based radiomic model that combined clinical and radiomic features to predict postoperative intermediary- and high-risk factors in patients undergoing surgery following neoadjuvant therapy. This combined model showed excellent diagnostic performance and can be potentially used for preoperative prediction of postoperative intermediary- and high-risk factors in patients with cervical cancer subjected to neoadjuvant therapy.

### Supplementary Information

Supplementary Information.

## Data Availability

The original contributions presented in the study are included in the article. Further inquiries can be directed to the corresponding authors.

## References

[1] Abu-Rustum NR, Yashar CM, Bean S (2020). NCCN guidelines insights: Cervical cancer, version 1.2020. J. Natl. Compr. Cancer Netw..

[2] Friedlander M, Kaye SB, Sullivan A (1983). Cervical carcinoma: A drug-responsive tumor—experience with combined cisplatin, vinblastine, and bleomycin therapy. Gynecol. Oncol..

[3] Gennigens C, De Cuypere M, Hermesse J, Kridelka F, Jerusalem G (2021). Optimal treatment in locally advanced cervical cancer. Expert Rev. Anticancer Ther..

[4] Tian X, Sun C, Liu Z (2020). Prediction of response to preoperative neoadjuvant chemotherapy in locally advanced cervical cancer using multicenter CT-based radiomic analysis. Front. Oncol..

[5] Sun C, Tian X, Liu Z (2019). Radiomic analysis for pretreatment prediction of response to neoadjuvant chemotherapy in locally advanced cervical cancer: A multicentre study. EBioMedicine..

[6] Miriyala R, Mahantshetty U, Maheshwari A, Gupta S (2022). Neoadjuvant chemotherapy followed by surgery in cervical cancer: Past, present and future. Int. J. Gynecol. Cancer..

[7] Chuang LT, Temin S, Camacho R (2016). Management and care of women with invasive cervical cancer: American society of clinical oncology resource-stratified clinical practice guideline. JGO..

[8] Gupta S, Maheshwari A, Parab P (2018). Neoadjuvant chemotherapy followed by radical surgery versus concomitant chemotherapy and radiotherapy in patients with stage IB2, IIA, or IIB squamous cervical cancer: A randomized controlled trial. JCO..

[9] Zeng J, Sun P, Ping Q, Jiang S, Hu Y (2022). Clinical outcome of FIGO 2018 stage IB3/IIA2 cervical cancer treated by neoadjuvant chemotherapy followed by radical surgery due to lack of radiotherapy equipment: A retrospective comparison with concurrent chemoradiotherapy. PLoS ONE..

[10] Tu H, Huang H, Ouyang Y (2020). Neoadjuvant chemotherapy followed by radical surgery versus concurrent chemoradiotherapy in patients with FIGO stage IIB cervical cancer: The CSEM 006 study. Int. J. Gynecol. Cancer..

[11] Gillies RJ, Kinahan PE, Hricak H (2016). Radiomics: Images are more than pictures they are data. Radiology..

[12] Wu G, Jochems A, Refaee T (2021). Structural and functional radiomics for lung cancer. Eur. J. Nucl. Med. Mol. Imaging..

[13] Conti A, Duggento A, Indovina I, Guerrisi M, Toschi N (2020). Radiomics in breast cancer classification and prediction. Semin. Cancer Biol..

[14] Bowen SR, Yuh WTC, Hippe DS (2017). Tumor radiomic heterogeneity: Multiparametric functional imaging to characterize variability and predict response following cervical cancer radiation therapy. J. Magn. Reson. Imaging..

[15] Chong GO, Park SH, Park NJY (2021). Predicting tumor budding status in cervical cancer using MRI radiomics: Linking imaging biomarkers to histologic characteristics. Cancers..

[16] Kan Y, Dong D, Zhang Y (2018). Radiomic signature as a predictive factor for lymph node metastasis in early-stage cervical cancer. J. Magn. Reson. Imaging..

[17] Chen X, Liu W, Thai TC (2020). Developing a new radiomics-based CT image marker to detect lymph node metastasis among cervical cancer patients. Comput. Methods Programs Biomed..

[18] Wu Q, Wang S, Chen X (2019). Radiomics analysis of magnetic resonance imaging improves diagnostic performance of lymph node metastasis in patients with cervical cancer. Radiother. Oncol..

[19] Giannini V, Pusceddu L, Defeudis A (2022). Delta-radiomics predicts response to first-line oxaliplatin-based chemotherapy in colorectal cancer patients with liver metastases. Cancers.

[20] Jeon SH, Song C, Chie EK (2019). Delta-radiomics signature predicts treatment outcomes after preoperative chemoradiotherapy and surgery in rectal cancer. Radiat. Oncol..

[21] van Dijk LV, Langendijk JA, Zhai TT (2019). Delta-radiomics features during radiotherapy improve the prediction of late xerostomia. Sci. Rep..

[22] Lin P, Yang PF, Chen S (2020). A delta-radiomics model for preoperative evaluation of neoadjuvant chemotherapy response in high-grade osteosarcoma. Cancer Imaging..

[23] Fiset S, Welch M, Weiss J (2019). Repeatability and reproducibility of MRI-based radiomic features in cervical cancer. Radiother. Oncol..

[24] Carter JV, Pan J, Rai SN, Galandiuk S (2016). ROC-ing along: Evaluation and interpretation of receiver operating characteristic curves. Surgery..

[25] Xiao M, Ma F, Li Y (2020). Multiparametric MRI-based radiomics nomogram for predicting lymph node metastasis in early-stage cervical cancer. J. Magn. Reson. Imaging..

[26] Fang M, Kan Y, Dong D (2020). Multi-habitat based radiomics for the prediction of treatment response to concurrent chemotherapy and radiation therapy in locally advanced cervical cancer. Front. Oncol..

[27] Hsu HC, Li X, Curtin JP, Goldberg JD, Schiff PB (2015). Surveillance epidemiology and end results analysis demonstrates improvement in overall survival for cervical cancer patients treated in the era of concurrent chemoradiotherapy. Front. Oncol..

[28] Kato T, Watari H, Takeda M (2013). Multivariate prognostic analysis of adenocarcinoma of the uterine cervix treated with radical hysterectomy and systematic lymphadenectomy. J. Gynecol. Oncol..

[29] Ren J, Li Y, Yang JJ (2022). MRI-based radiomics analysis improves preoperative diagnostic performance for the depth of stromal invasion in patients with early stage cervical cancer. Insights Imaging..

