# Supplementary material for: Delta radiomics analysis for prediction of intermediary- and high-risk factors for patients with locally advanced cervical cancer receiving neoadjuvant therapy
Source: Sci Rep. 2023 Nov 8;13:19409. doi: 10.1038/s41598-023-46621-y (PMC10632513; doi:10.1038/s41598-023-46621-y)
Supplement: Supplementary file 1 — Supplementary Information. [file 41598_2023_46621_MOESM1_ESM.docx]

The radiomics model contains the following 18 features:

gradient_glcm_DifferenceVariance、

log-sigma-3-0-mm-3D_glcm_ClusterShade、

exponential_glrlm_RunPercentage、

wavelet-LLH_glcm_JointEntropy、

wavelet-HLH_glcm_DifferenceAverage 、

wavelet-LHL_glcm_ClusterTendency 、

gradient_glcm_ClusterShade、

wavelet-LHL_glcm_Idn、

wavelet-LHH_firstorder_RobustMeanAbsoluteDeviation、

wavelet-LHL_glcm_DifferenceVariance 、

wavelet-HLL_firstorder_Kurtosis 、

log-sigma-5-0-mm-3D_glcm_Autocorrelation、

wavelet-HLH_glcm_Idm 、

wavelet-HLH_firstorder_10Percentile 、

original_shape_Flatness 、

log-sigma-5-0-mm-3D_glcm_DifferenceAverage 、

gradient_glcm_JointAverage、

log-sigma-5-0-mm-3D_glcm_JointAverage .

The combine model contains the following 19 features: Diameter_more_than_4cm、

wavelet-LHL_firstorder_Maximum、

Pre_SCC、

log-sigma-3-0-mm-3D_glcm_ClusterShade、

gradient_glcm_ClusterShade、gradient_firstorder_Range、

Maximum_tumor_diameter、

square_glcm_ldm、

log-sigma-2-0-mm-3D_glcm_ldn、

wavelet-LHL_firstorder_Range、

log-sigma-2-0-mm-3D_firstorder_Skewness、

log-sigma-5-0-mm-3D_glcm_lmc1、

Neo_Scc、

squareroot_glcm_DifferenceVariance、

wavelet-HLH_glcm_JointEntropy exponential_glcm_ClusterShade、

square_firstorder_Range

Recist1.1_Withdrawal_rate、

log-sigma-5-0-mm-3D_glcm_JointAverage.
